# Dispensing and determinants of non-adherence to treatment for non complicated malaria caused by *Plasmodium vivax* and *Plasmodium falciparum* in high-risk municipalities in the Brazilian Amazon

**DOI:** 10.1186/s12936-015-0998-3

**Published:** 2015-11-26

**Authors:** Claudia G. S. Osorio-de-Castro, Martha C. Suárez-Mutis, Elaine S. Miranda, Tatiana C. B. Luz

**Affiliations:** Núcleo de Assistência Farmacêutica, Escola Nacional de Saúde Pública Sérgio Arouca, Fundação Oswaldo Cruz, Rua Leopoldo Bulhões 1480, sala 625, Manguinhos, Rio de Janeiro CEP 21041-210 Brazil; Laboratório de Doenças Parasitárias, Instituto Oswaldo Cruz, Fundação Oswaldo Cruz, Av Brasil 4365. Pav Artur Neiva. Sala 21B, Manguinhos, Rio de Janeiro CEP 21040-900 Brazil; Departamento de Farmácia e Administração Farmacêutica, Faculdade de Farmácia, Universidade Federal Fluminense, Rua Mário Viana, 523, CEP 24241-000 Niterói, RJ Brazil; Laboratório de Educação em Saúde e Ambiente, Centro de Pesquisa René Rachou, Fundação Oswaldo Cruz, Belo Horizonte, MG Brazil

**Keywords:** Malaria, Brazil, Dispensing, Adherence, Medicines

## Abstract

**Background:**

In Brazil, 99.7 % of malaria cases occur in the Amazon region. Although the number of cases is decreasing, the country accounted for almost 60 % of cases in the Americas Region, in 2013. Novel approaches for malaria treatment open the possibility of eliminating the disease, but suboptimal dispensing and lack of adherence influence treatment outcomes. The aim of this paper is to show the results on dispensing practices, non-adherence and determinants of non-adherence to treatment of non-complicated malaria.

**Methods:**

The study was conducted in six high-risk municipalities with *Plasmodium vivax* and *Plasmodium falciparum* transmission in the Brazilian Amazon and based on the theoretical framework of the Mafalda Project, which included investigation of dispensing and adherence. The World Health Organization Rapid Evaluation Method has been used to estimate sample size. Individuals over 15 years of age with malaria were approached at health facilities and invited to participate through informed consent. Data was collected in chart review forms focusing on diagnosis, *Plasmodium* type, prescribing, and dispensing (kind, quantity, labelling and procedures). Follow-up household interviews complemented data collection at health facility. Non-adherence was measured during the implementation phase, by self-reports and pill-counts. Analysis was descriptive and statistical tests were carried out. Determinants of non-adherence and quality of dispensing were assessed according to the literature.

**Results:**

The study involved 165 patients. Dispensing was done according to the national guidelines. Labelling was adequate for *P. vivax* but inadequate for *P. falciparum* medicines. Non-adherent patients were 12.1 % according to self-reports and 21.8 % according to pill-counts. Results point to greater non-adherence among all *P. falciparum* patients and among malaria non-naîve patients. More patients informed understanding adverse effects than ‘how to use’ anti-malarials.

**Conclusions:**

Non-adherent patients were mostly those with a *P. falciparum* diagnosis and those in their second or more malaria episode. New taxonomies and concepts on adherence stress the importance of focusing on the individual patient. Interventions targeted to and tailored for malaria patients must be addressed by health policy and implemented by managers and clinicians.

## Background

Despite the decrease in the number of malaria cases worldwide in the past decade, this disease remains a major public health problem. Brazil accounted for almost 60 % of cases in the Americas Region, with 178,613 cases reported in the year 2013. Transmission still occurs in 808 municipalities of the country [1, 2]; 99.7 % of cases occur in the Amazon region where *Plasmodium vivax* and *Plasmodium falciparum* co-exist, and to a lesser extent, *Plasmodium malariae* is present.

Following the recommendations of the Amsterdam conference in 1992, Brazil bases malaria control on early diagnosis and adequate treatment, while also favouring prevention strategies [3]. For *P. vivax* malaria, for instance, the recommended treatment is the combination of chloroquine plus primaquine and this protocol remains unchanged.

In 2006, the Brazilian National Malaria Control Programme (NMCP) introduced changes in *P. falciparum* malaria treatment, from quinine plus doxycycline plus primaquine to artemisinin combination therapy (ACT). According to NMCP, this treatment change resulted in a decrease in total number of *P. falciparum* cases, from 24.9 % of all registered malaria cases in 2006, to <16.2 % in 2011 [1]. Novel approaches for malaria open the possibility of eliminating the disease, in specific situations [4]. In this context the correct use of anti-malarials is tantamount for disease control. This is one additional reason for studying treatment-based control policies.

Lack of treatment adherence is frequent in malaria [5], due to a number of factors that may be present, such as lack of prescription or written instructions, regimen complexity, adverse effects [6]. Sub-optimal dispensing is an important service determinant that may also be present and influence treatment outcomes. These may lead to intermittent dosing and eventually to drug resistance [7].

Adherence has been measured by a series of direct and indirect methods, and is usually expressed as a percentage of total number of doses, according to dosing regimen [6, 8, 9]. Controlled studies have recently characterized adherence as a multi-step or multi-phase process, involving initiation (or decision to start dosing), implementation (actual dosing history) and discontinuation (cessation) of the treatment regimen. The maintenance of treatment after initiation and before discontinuation is called persistence [7, 8]. In field conditions adherence, or non-adherence, may be investigated by questionnaires to produce estimated measures such as self-reports or pill counts. Both of these methods measure implementation and may clarify essential details regarding this phase, even if not other phases [10]. Overall, adherence remains an essential factor for malaria patients and although considerable work has been done to assess magnitude of adherence, several questions regarding adherence remain unanswered [11].

The main goal of this study is to show the results on dispensing practices, non-adherence to treatment during the implementation phase and determinants of non-adherence to treatment of non-complicated malaria in settings with *P. vivax* and *P. falciparum* transmission in high-risk municipalities of the Brazilian Amazon.

## Methods

The methods employed in this work were based on the theoretical framework of the “Mafalda” Project (“Pharmaceutical services for non-complicated malaria by *P. vivax* and *P. falciparum* in high-risk municipalities of the Brazilian Amazon: organization of services, prescribing, dispensing and adherence to treatment”). Initially, in order to subsidize the project, a comprehensive review of pharmaceutical services for malaria was carried out and published [12]. The framework comprising a logic model and 25 indicators was developed and subsequently published [13]. The indicators included in the framework encompassed the following dimensions: context and organization of pharmaceutical services, prescribing, dispensing and (implementation) adherence to treatment. The first two dimensions were developed and published [14]. This paper will focus on the implementation phase and examine non-adherence, by means of a careful examination of determinants, which include those linked to treatment regimen and those linked to health services and care to malaria patients, including dispensing practices.

The method was based on World Health Organization (WHO) guidelines [15] and adapted by Management Sciences for Health (MSH) [16], in which evaluations of non-complicated malaria require a sample of not under 600 patient registries (investigated at health facility level) is recommended [17], complemented by at least 150 patients at household level.

### Data collection instruments

Questionnaires, observation forms, interviews forms and chart review forms were used during field work, according to the dimension that was being investigated [13]. Specific forms, for quantitative and qualitative data, were used for organization of services, prescribing and dispensing. A chart review form compiled data on patient diagnoses, type of *Plasmodium* spp, treatment characteristics (including prescribing), dispensing (dispensing process, medicines received, in kind and quantity, labelling), information given to patient by health worker (regarding use of medicines, adverse effects and how to keep medicine at home) and compliance to national guideline. For the household survey, another data collection instrument, for objective data, was applied, according to treatment regime.

### Field study

The investigation was carried out in six malaria high-risk municipalities of the Brazilian Amazon, selected according to Annual Parasitic Index (API) and population. Up to four high-coverage primary health care facilities were selected to guarantee number of eligible individuals. Individuals of both genders, ≥15 years of age, excluding pregnant women, with parasitological confirmed mild malaria were followed throughout the study. More detailed information on the field study and participants may be found elsewhere [14].

All procedures for investigation of organization of services and prescribing were complemented by those designed to investigate dispensing and adherence. For information regarding these dimensions, patients were approached in a two-step process. Data collection during consultation at health facility and observation of dispensing practices was complemented by household interviews. Patients that had been recruited for the first part of the investigation (consultation at health facility) were asked if household follow-up visit was welcomed, which indicated their inclusion in the next part of the investigation. Data was collected, according to *P. falciparum* or to *P. vivax* treatment, on the second or on the fifth day of treatment (D2 or D5), respectively.

### Analysis

Analysis was based on the theoretical framework developed for the Mafalda Project [13]. Dispensing was characterized by drugs dispensed according to prescription or indication, information given to patient during dispensing; adequate labelling of medicines; patients reporting knowledge of treatment regimen and adverse effects. Adequacy of labelling was assessed by direct observation during dispensing of treatment regimens at health facilities.

Adherence was approached considering the assumption that all patients initiated treatment on D0 and discontinued treatment at the end of D2 (*P. falciparum*) or D5 (*P. vivax*). Household visits occurred during the last day of treatment. Non-adherence was measured during the implementation phase, by self-reports (adherence accepted as no missed doses during treatment period) and pill counts (adherence accepted as quantity received as proxy of quantity consumed), both of which are expressed by percentages.

Determinants of non-adherence related to quality of dispensing were evaluated according to in-place requirements at facilities suggested by the literature [6, 7, 9, 18, 19]. Other determinants related to disease (diagnosis, first malaria episode, general well-being in the present malaria episode) patient characteristics and treatment characteristics (first-line treatment, adverse effects, use of other medicines, care-seeking behaviour) were analysed according to adherence group. Among non-adherent individuals, reasons given for non-adherence during household visit were described.

### Statistical analysis

A simple test of differences between proportions was carried out to investigate possible discrepancies in results, admitting a 95 % CI [20]. Concordance between measures of adherence (patient reports and pill counts) was carried out by calculation of the *kappa* index [21].

### Ethical considerations

All participants were asked to sign an informed consent form and furthermore to give written agreement for household visits. The Sergio Arouca National School of Public Health, Oswaldo Cruz Foundation (Fiocruz) Ethics in Research Committee gave study approval (Approval number 91/06; CAAE 0086.0.031.000.06).

## Results

### Dispensing

Treatment regimen was examined for 165 adult patients. This number was 10.0 % over the minimum number of patients required for the study of adherence as described in the study design. Of the 165 patients, 134 (81.2 %) had been diagnosed with *P. vivax,* all receiving first-line treatment (Table 1) and 31 with *P. falciparum*, of which 16 (51.6 % of *P. falciparum* patients) had received first-line treatment at the time of the study (Table 1). Twenty-eight patients (16.9 % overall) were experiencing their first malaria infection.

**Table 1:** Diagnoses and therapeutic regimens dispensed per malaria patient

Type of malaria	Therapeutic regimen	n (%)
*P. vivax*	Chloroquine and primaquine	134 (81.2)
*P. falciparum*	Quinine sulfate, doxycycline and primaquine	16 (9.7)
	Artemether–lumefantrine and primaquine	14 (8.5)
	Mefloquine and primaquine	1 (0.6)
Total		165 (100)

Mafalda project, 2007

Observation of dispensing for these patients in health facilities first resulted in findings related to dispensing requirements. Dispensing of first-line regimens followed indication, and was done in accordance to the national guideline, for *P. vivax* as well as for *P. falciparum*. Among 165 patients, 134 patients (81.2 %) received verbal instruction at dispensing; 112 (67.9 %) patients informed understanding of how to use the anti-malarials and 137 (83 %) of their possible adverse effects.

In regard to labelling of medicines at dispensing, shortcomings related to name of medicine, dose or dosage form were observed. Labelling of chloroquine and primaquine were adequate in 80 % of *P. vivax* regimens. At the time of data collection, first-line regimen for *P. falciparum* included quinine sulfate, doxycycline and primaquine. However, labels for these medicines (quinine, n = 16, doxycycline, n = 16 and primaquine, n = 16) were all inadequate. Labels presented problems among the 15 patients receiving alternative *P. falciparum* treatment, of which 14 received artemether–lumefantrine and primaquine. Inadequately labelled medicine was also dispensed to one patient receiving mefloquine and primaquine for *P. falciparum* (Table 1).

### Adherence and non-adherence

One hundred and sixty-five patients were visited for household interviews during investigation of adherence (134 *P. vivax* patients on Day 5 and 31 *P. falciparum* patients on Day 2). Non-adherence during implementation measured by self-reports revealed 144 adherent patients (there was one missing measure for among *P. vivax* patients). Twenty patients (12.2 %), non-adherent in self-reports, informed they had stopped using at least one anti-malarial during treatment (Table 2).

**Table 2:** Non-adherence to anti-malarial treatment by self-reports and pill counts

Characteristic	Non-adherence to anti-malarial treatment			
	Pill counts		Self-reports	
	n (%)	p value	n (%)	p value
Diagnosis				
*P. vivax*	24 (17.9)	0.012	12 (9.1)	0.014
*P. falciparum*	12 (38.7)		8 (25.0)	
First malaria episode				
Yes	2 (7.1)	0.044*	2 (7.1)	0.532*
No	34 (24.8)		18 (13.3)	
General well being in the present malaria episode				
Felt well/same as always	14 (20.6)	0.843	8 (11.8)	0.971
Felt bad	21 (21.9)		11 (11.6)	
First-line treatment				
Yes	35 (23.3 %)	0.195*	19 (12.8 %)	0.697*
No	1 (6.7 %)		1 (6.7 %)	
Adverse effects				
Yes	21 (22.1)	0.917	11 (11.7)	0.823
No	15 (21.4)		9 (13.0)	
Uses other medicines				
Yes	12 (22.6)	0.860	6 (11.3)	0.813
No	24 (21.4)		14 (12.6)	
Seeked professional care during treatment				
Yes	4 (25.0)	0.749*	4 (25.0)	0.096*
No	31 (20.9)		15 (10.2)	

Mafalda project, 2007

* Fischer’s exact test

Pill counts were conducted for 165 patients, and the quantity that they had in their possession, in relation to day of treatment, was accurate in 129 (78.2 %) and inaccurate for 36 (21.8 %), patients designated as non-adherent (Table 2). Concordance (*kappa*) between these two methods of measuring adherence was 0.74.

Table 2 also shows non-adherence determinants among individuals. *P. falciparum* patients were more prone to being non-adherent. This finding was significant for both measures of adherence implementation. Also significant for implementation non-adherence according to pill count were results for non-naive malaria patients (p = 0.012). All other variables were non-significant.

Regarding *P. falciparum* patients, of the 12 non-adherent by pill-counts, 11 were on the first-line regimen and one was on artemether–lumefantrine and primaquine; of the eight non-adherent patients by self-reports, seven were on first-line treatment and one was on the alternative regimen with artemether–lumefantrine and primaquine. The distribution of non-adherence determinants by malaria type was not possible because of sample size.

Various possible reasons for non-adherence were given by individuals: forgetfulness (8 interviewees), occurrence of adverse effects (10), unwillingness to take medicine (6), alcohol consumption (1), ‘felt cured’ (3), other (20). Patients sometimes informed one, two or more reasons for non-adherence.

## Discussion

The Mafalda Project (“Pharmaceutical services for non-complicated malaria by *P. vivax* and *P. falciparum* in high-risk municipalities of the Brazilian Amazon: organization of services, prescribing, dispensing and adherence to treatment”) was developed in order to provide data of the situation of pharmaceutical services for malaria in municipalities at high risk for the disease in the Amazon [13, 14, 22, 23].

Previous results from the Mafalda Project have shown that in Brazil diagnostic procedures work well, but good prescribing practices are not performed in most municipalities. Other findings showed problems with organization of pharmaceutical services—especially concerning stock management and drug storage [14]. There are no written instructions for the malaria patient and only oral guidance is received from technicians in endemic areas. Few qualified professionals (physicians) actually prescribe [23]. Health workers have little formal education while training is informal or insufficient [23]. In this context it seems difficult that these workers can effectively contribute to the processes that lead to adherence [6].

This study focused specifically on dispensing and adherence. Main findings in this study showed that dispensing was carried out according to the national guidelines. A greater proportion of patients informed understanding adverse effects over ‘how to use’ anti-malarials. Labelling was adequate for *P. vivax* but inadequate for *P. falciparum* medicines. Self-reports accounted for 144 adherent patients and pill counts for 129.

Well-reported studies in the Brazilian Amazon have shown differences in adherence. One measured adherence of *P.vivax* patients by a standardized scale and presented similar results as to range of non-adherence, but an overall higher proportion of non-adherent patients—33.3 % [9]. Other studies showed lower percentages of non-adherent individuals: 9.6 % for non-adherent *P. vivax* patients in Pará State [24] and 16 % non-adherence for *P. vivax* and *P. falciparum* patients in Mato Grosso [25].

This difference between methods and problems with questionnaire consistency has been described previously in the literature [6] and may account for the discrepancy. In this case, concordance between methods was 0.74. There was more implementation non-adherence among *P. falciparum* patients and among non-naive patients. Various previous malaria episodes in the same patient may be a barrier to complete treatment. Patients usually discontinue medication as they feel better and malaria-savvy patients may disregard need to finalize treatment regimen [26]. Adequate dispensing with assertive information as to the risks of not completing the treatment might have had a positive influence on these patients.

It is noteworthy to mention that lack of adequate labelling for *P. falciparum* medicines coincided with greater non-adherence for this group of patients. In regions where many patients are illiterate, family members who can read may provide support for better understanding of treatment regimens by patients, at least by reading the labels and instructions on how to use their medicines. During the Mafalda Project, ACT had been introduced by PNCM in certain areas of the Amazon but were labelled in English. This evidently worsened the situation. Recently this is undergoing change. Anti-malarials are now supplied in blisters labelled with symbols for better understanding [27]. Lack of written instructions for medicines, however, persists.

A greater number of patients (83 %) mentioned understanding adverse effects. These possible malaria treatment outcomes may be acutely felt by patients [23], and are therefore valued as a worthwhile reason for non-adherence to treatment, as reported by ten individuals (27.8 %). Drugs with hazardous effects, even on the first dose, such as some anti-malarials, may cause ‘off–on’ episodes in treatment implementation, which account for abrupt changes to drug exposure, compromising treatment response and fostering resistance [28, 29].

Results point to greater non-adherence among all *P. falciparum* patients and among malaria non-naive patients. For the first group, reasons may be associated to change in treatment regimens, from a three-medicine 5-day treatment to a single-medicine (ACT) 3-day treatment. Many patients experienced various episodes of *P. falciparum* malaria and were already accustomed to the treatment regimen. With the lack of adequate instructions and labels, switches might be confusing. Another possibility is the acuteness of adverse effects with the traditional *P. falciparum* regimen (quinine sulfate, doxycycline, primaquine), leading patients to discontinue treatment as soon as they feel a little better.

More than suboptimal dosing history (implementation of treatment regimen), early discontinuation (or short persistence) is the largest single factor for a decrease in adherence [7]. Three patients mentioned ‘feeling cured’ as a reason for early discontinuation. Individuals who experience malaria for the first time are apt to feel fear and recur to treatment, while those who have had more than one episode may feel more confident and not so treatment-dependent. Fourteen non-adherent patients were forgetful and-or unwilling to take their medicines. However, a precise understanding of discontinuation was not possible, due to methodological limitations. Visits were conducted at the end of treatment but actual treatment discontinuation was not observed or measured.

Adherence is a crucial step for any pharmacological treatment. Acute-phase, complex treatments, such as anti-malarial treatment, oblige prescriber-patient collaboration mainly as to the initiation and implementation steps of adherence [8]. Interventions, such as the NMCP, may not be sufficient to secure adherence. Population-targeted approaches would need to be developed for non-adherent individuals, while tailored approaches would need to focus on the principal causes and determinants for non-adherence [28]. As such, these steps must begin to be addressed by health managers and clinicians. Relevant information on non-adherence by malaria patients is essential for health-based interventions that aim to decrease therapeutic failure and emergence resistance for *P. falciparum* and *P. vivax*.

*Plasmodium falciparum* and non-naïve patients constitute possible target groups for adherence interventions in malaria treatment [28]. Brazil has a low number of *P. falciparum* cases [1] and the goal is to eliminate *P. falciparum* malaria before emergence of resistance to ACT [4]. However, this may prove to be difficult given the context in the Amazon. As such, alternative and concurrent strategies [29] must be taken to improve treatment effectiveness and retard resistance emergence. One of these strategies is improving adherence in all its stages (initiation, implementation and discontinuation) [8] and identifying non-adherent patients. Tailoring interventions for individual non-adherent patients, on the other hand, is only possible through understanding the determinants associated with non-adherence [18, 28].

Studies on adherence cover many types of definitions of adherence and measures [8, 9]. Several employ self-reports or pill counts, or both, in order to improve validity. This study presented both methods and concordance between them of 0.74, considered good [21]. Nonetheless, both methods may overestimate adherence—or underestimate non-adherence.

Concepts on adherence have traditionally followed a stepwise process. The WHO [19] has proposed five groups of adherence determinants—linked to health system, to disease, to the individual, to social-economic aspects, and to treatment-related aspects. Other authors [18, 30] have also studied adherence-related determinants. Apart from variables associated with the various determinants of adherence, Kardas and colleagues [18] point out that actual organizational processes—correct prescribing and adequate dispensing—have a direct impact on adherence and can invalidate control efforts (and in case of *P. falciparum*, elimination). This may be the case with malaria, mainly because of complex treatment regimens [6, 30].

Through controlled studies, new concepts associated with adherence have emerged which subvert previous understanding of how adherence should be measured [6, 8–10, 28]. This has consequences on how to design studies on adherence and on how limited may findings such as ours be on actually measuring adherence in a given population. This study is limited to implementation adherence and in that to overall percentages of implementation, not being able to acknowledge the actual links between prescribing and drug dosing histories, so well put by Blaschke and colleagues [7]. Nevertheless, by acknowledging information gathered on overall implementation non-adherence and its determinants, results may shed light on needs for policy interventions, such as close patient monitoring and preventive measures to curb lack of treatment effectiveness.

As the sample in this study was designed for a traditional measure of adherence, numbers produced an overall idea of adherent and non-adherent patients, in respect to treatment implementation. Determinants for non-adherence were consistent with the literature [10]. However, non-adherence caused by sub-optimal initiation or discontinuation could not be identified by this approach. The small sample also impeded us from distinguishing between non-adherent *P. falciparum* patients in respect to differences in treatment regimens.

## Conclusions

This study produced an overview of non-adherence measured in the implementation phase and of determinants associated with non-adherence among *P. falciparum* and *P. vivax* patients in the Brazilian Amazon. Non-adherent patients were mostly those with a *P. falciparum* diagnosis and those in their second or more malaria episode. In face of new taxonomies and concepts on adherence and because the emergence of resistance to ACT and other anti-malarials are of utmost importance to public health, interventions targeted to and tailored for malaria patients must be addressed by health policy and implemented by managers and clinicians.
